# 一项在欧洲和中国进行的新型胃泌素释放肽前体（ProGRP）免疫检测多中心评估研究

**DOI:** 10.3779/j.issn.1009-3419.2017.08.12

**Published:** 2017-08-20

**Authors:** Catharina M. KORSE, Stefan HOLDENRIEDER, Xiuyi ZHI, Xiaotong ZAHNG, Ling QIU, Andrea GEISTANGER, Marcus-Rene LISY, Birgit WEHNL, Daan van den BROEK, José M. ESCUDERO, Jens STANDOP, Mu HU, Rafael MOLINA

**Affiliations:** 1 The Netherlands Cancer Institute, Plesmanlaan 121, Amsterdam 1066CX, The Netherlands; 2 University Hospital Bonn and Center of Integrative Oncology Cologne/Bonn, Sigmund-Freud-Strasse, Bonn 25-53127, Germany; 3 Xuanwu Hospital, Capital Medical University, No. 45 Changchun Street, Xicheng District, Beijing 100053, China; 4 Peking Union Medical College Hospital (PUMCH), 1Shuaifuyuan, Dongcheng District, Beijing 100730, China; 5 Roche Diagnostics GmbH, Nonnenwald 2, Penzberg 82377, Germany; 6 Hospital Clinic, University of Barcelona, Carrer Villarroel 170, Barcelona 08036, Spain

**Keywords:** 鉴别诊断, 免疫检测, 促胃泌素释放肽前体, ProGRP, SCLC, 稳定性

## Abstract

**背景:**

在欧洲和中国进行Elecsys^®^胃泌素释放肽前体（ProGRP）免疫检测的多中心评估研究。

**方法:**

在欧洲的3个中心和中国的2个中心，在肺癌中，通过不精密度、稳定性、方法学比较和鉴别诊断能力来评价该检测法。

**结果:**

5个分析物浓度的中间不精密度范围为变异系数：2.2%-6.0%。在不同储存条件下，血浆和血清样本均显示出良好的稳定性。在血浆中Elecsys^®^和ARCHITECT检测（斜率1.02，截距-2.72 pg/mL）之间表现出良好的相关性。同时，Elecsys^®^检测在血清和血浆样本之间表现出良好的相关性（斜率0.93，截距2.35 pg/mL；相关系数0.97）。ProGRP作为不受种族、年龄、性别或吸烟史相关影响的检测手段，可鉴别小细胞和非小细胞肺癌（NSCLC）；截断值为84 pg/mL时，曲线下面积为0.90，95%CI: 0.87-0.93；敏感性为78.3%，特异性为95%。ProGRP浓度中位数在良性病变（38 pg/mL）、其他恶性肿瘤（40 pg/mL）或NSCLC（39 pg/mL）中较低，而在3期以上慢性肾脏疾病中浓度较高（>100 pg/mL）。

**结论:**

Elecsys^®^ ProGRP检测在血清和血浆中稳定性增加，较现有检测法明显更具优势。ProGRP检测在中国的首次评价在不同种族中显示出相当的鉴别能力。

## 1 前言

肿瘤标志物作为肺癌两种主要亚型[非小细胞肺癌（non-small cell lung cancer, NSCLC）和小细胞肺癌（small cell lung cancer, SCLC）]的鉴别手段，已经在肺癌患者中得到广泛研究，进而改善了诊断和治疗选择^[1-5]^。NSCLC占所有新发肺癌病例的80%左右，SCLC占20%左右。SCLC不同于NSCLC，其具有神经内分泌分化、较高肿瘤生长速度和较早转移^[6, 7]^，因此需要采取不同的治疗方法。在这两种亚型中，NSCLC更有可能在早期被诊断，此时手术可以提供最好的治愈机会^[8]^。SCLC的早期诊断非常少见，意味着手术并非常见治疗手段，但其对放射治疗和化疗高度敏感^[7]^。SCLC患者常复发，然而近年来，其5年生存率保持不变^[9]^。初诊时，肺癌亚型的鉴别诊断对于确保采取适当治疗干预至关重要。

肿瘤活检是肺癌组织学鉴别诊断的重要组成部分。然而，由于许多SCLC存在于粘膜下层，组织精确取样可能存在困难，所以活检无法帮助疾病早期发现^[10]^。当被诊断为处于疾病局限期时，约20%的SCLC患者可以通过接受积极化学治疗和放射治疗实现长期生存率，而确诊为晚期时，仅为5%^[10]^。血浆或血清样本中肿瘤标志物的分析较组织学鉴别诊断明显更具优势，包括发现早期SCLC的能力，以及提高生存率的机会。

神经元特异性烯醇化酶（NSE）和胃泌素释放肽前体（ProGRP）已被证明是SCLC最有价值的肿瘤标志物^[11, 12]^。虽然长久以来，NSE作为被推荐的SCLC肿瘤标志物^[13]^，在组织检查中NSE也可以染色高达80% NSCLC组织，但仅20%-30% NSCLC患者的血清中NSE升高^[14]^。此外，NSE敏感性较低，特别是对于病变局限于一侧胸腔或同侧纵隔的患者^[15]^。由于NSE存在于血小板和红细胞中，因此必须排除溶血样本，并且样本的快速储存至关重要^[14]^。ProGRP可准确鉴别NSCLC和SCLC^[16, 17]^，ProGRP浓度在其他恶性疾病或良性病症中很少升高，而在肾功能不全、肺神经内分泌肿瘤（NET）和甲状腺髓样癌（MCT）患者中表现高浓度^[16-22]^。

在2009年，报道了首个全自动化ProGRP ARCHITECT检测（Abbott Laboratories, Wiesbaden, Germany）的评估^[23]^。由于在ARCHITECT检测中，ProGRP在血清中稳定性差，研究认为原因是凝血酶诱导的蛋白水解，因此将血浆样本作为推荐的源材料^[24, 25]^。Elecsys^®^ ProGRP检测（Roche Diagnostics GmbH, Penzberg, Germany）是一种新的免疫检测法，旨在定量测定人血清和血浆中ProGRP的水平。由于Elecsys^®^ ProGRP检测中的两种单克隆抗体与相对耐内切蛋白酶酶切的ProGRP肽表位结合^[16, 17, 26]^（补充图S1），因此可以使用血清样本以及血浆样本。在本文中，我们报告了欧洲和中国多个中心的Elecsys^®^ ProGRP检测的技术和临床性能。

## 2 材料和方法

2012年8月-2013年9月期间，在3个欧洲研究中心（Amsterdam、Barcelona和Bonn）和北京的2个中国研究中心[北京协和医院（PUMCH）和北京宣武医院]对Elecsys^®^ ProGRP检测进行了评估。在临床研究工作开始之前，从各机构获得了伦理批准/豁免。所有研究中心均遵照赫尔辛基宣言（修订版Tokyo、Venice、Hong Kong和Fortaleza 2013）和国际协调会议关于临床试验管理规范进行研究。该研究的目的是评估Elecsys^®^ ProGRP检测在不精密度、稳定性、方法学比较和SCLC鉴别诊断能力方面的性能。

### 2.1 检测说明

Elecsys^®^ ProGRP检测是一种电化学发光免疫检测，其使用生物素化的ProGRP特异性小鼠单克隆抗体和钌标记的ProGRP特异性小鼠单克隆抗体捕获和检测人血清和血浆中的ProGRP。该检测采用ProGRP CalSet（Roche Diagnostics）进行定标，并可溯源至ARCHITECT ProGRP检测。使用两个水平的PreciControl ProGRP（Roche Diagnostics）进行质量控制。定标液和质控液均包含重组ProGRP。

### 2.2 样本来源、制备和处理

样本均来自先前未经治疗的活动性病变患者，其具有足够的样本用于分析。未进一步使用人口学或组织学选择标准。肺癌组织学分类根据1999年世界卫生组织指南^[27]^。SCLC和NSCLC的鉴别诊断基于形态学特征以及肿瘤CD56阳性和/或突触素免疫组化。根据国际指南确定肺癌分期（TNM）^[28]^。

所有研究中心均采集样本并进行检测。另外一个德国中心（Institut für Klinische Pharmakologie GmbH, Kiel）提供了来自表观正常人群的样本，作为参考队列。由于SCLC在患者群体中的患病率低，鉴别诊断主要基于来自欧洲样本库的患者血清样本，而在中国样本为前瞻性采集（2013年1月-9月）。

所有3个欧洲中心和PUMCH均使用血清进行临床评价。宣武医院使用EDTA-K2原始管进行血浆采集。在Bonn和Amsterdam使用EDTA-K2和血清分离管（SST）进行稳定性实验。此外，Amsterdam在本实验中使用快速血清管（RST）进行血清采样。RST的内壁涂有凝血酶，以促进快速凝血。所有研究均在cobas^®^ e411和e601分析仪上进行。

### 2.3 统计分析

使用WinCAEv（基于Windows的计算机辅助评价）软件程序采集所有分析数据。使用MACRO软件程序收集人口统计学和临床资料。在Roche Diagnostics Penzberg的生物统计学部门采用SAS（统计分析软件9.2版）和R（版本2.13.2）进行所有使用临床信息的统计分析。对于目视检查主数据集确定的异常值，进行重新测定。

### 2.4 技术评估

#### 2.4.1 实验室间调查

通过Roche R&D（-30 pg/mL、-200 pg/mL和-1, 500 pg/mL）制备3个EDTA血浆样本池，并分发到所有5个研究中心，以评估这3个浓度范围内实验室间回收率和日间变异性差异。同时，PreciControl ProGRP的两个水平也被用作样本材料。在各实验室中，10天内单次测定每个样本。每个样本的回收率百分比计算为测定浓度/所有实验室中位数×100。

#### 2.4.2 根据临床实验室标准协会EP5-A2的不精密度

通过临床实验室标准协会（CLSI）EP5-A2指南^[29]^评估不精密度。由欧洲中心制备的三个样本池的目标浓度范围分别为7 pg/mL-60 pg/mL、61 pg/mL-1000 pg/mL和1, 001 pg/ mL-5, 000 pg/mL。取约27 mL的血清或血浆，制备84×300 μL等分试样。将样本在-20 ℃下储存，并在相应的测定日使用。通过根据CLSI EP5-A2方差分量模型对数据进行建模，获得了重复性和中间精密度估值。以日间、批间和重复性方差分量进行建模。基于总方差估计，给出了中间精密度估值，作为变异系数（CV）。

#### 2.4.3 样本材料的稳定性

经机构审查委员会批准，从提供知情同意书的患者处获得相应的血清和血浆样本。理想状态下，样本中ProGRP的浓度超过100 pg/mL；然而，由于获得知情同意书和样本存在困难，本研究纳入了一些低于此浓度的样本。至少从每位患者体内获取3 mL血清和3 mL血浆，以制备9份300 μL等分试样。在取样1 h内进行测定，作为基线测定值，并室温下1 h、2 h、3 h和4 h后，以及在2 ℃-8 ℃下3 h、6 h、24 h和48 h分别进行测定。首先在ARCHITECT仪器上进行测定，然后在30 min内，在cobas^®^仪器上进行测定。在同一批次中检测血清和血浆。回收率百分比计算为实际浓度/基线浓度×100。

#### 2.4.4 方法学比较

使用ARCHITECT ProGRP检测和Fujirebio微量滴定板酶联免疫吸附剂ProGRP检测（Fujirebio Diagnostics, Japan）获得比较结果。理想情况下，血浆样本应用于ARCHITECT仪器，以及血清样本用于Fujirebio检测。误差率为1的Deming回归用于估计回归线^[30]^。通过Bootstrap方法获得截距和斜率的置信区间（CI）^[31]^。

#### 2.4.5 矩阵比较

从2012年第三季度-2013年第一季度的常规取样收集了匹配的血清和血浆标本，并在2013年第一季度冷冻至分批测定。数周采集样本以涵盖整个测量范围（3 pg/mL-5, 000 pg/mL）。

## 3 临床评估

### 3.1 参考范围确定

对于ProGRP参考范围计算，根据基于对健康检查问卷的回答以及葡萄糖、胆碱酯酶、肌酐、C-反应蛋白和血红蛋白水平等基础临床化学参数的结果，在来自5个欧洲中心和2个中国中心的健康人群队列，以及从K iel中心招募的表观正常人群队列中进行比较。在K iel中心采集了三种样本类型：血清、EDTA-K2血浆和肝素锂血浆。来自Kiel的样本在-80 ℃下储存于Roche Penzberg样本库中，并在Bonn进行测定。Amsterdam、Barcelona和Bonn仅采集血清样本。中国PUMCH采集血清和EDTA-K3血浆样本，而宣武医院仅采集EDTA-K2血浆样本。在R软件中使用类型=3方法计算分位数。关于Hahn和Meeker的方法，CI是非参数^[32]^。

### 3.2 鉴别诊断

NSCLC和SCLC恶性病变队列是主要焦点，表观正常人群、肺部良性病变患者（如慢性阻塞性肺病、结核、肺炎和哮喘）、其他良性疾病患者（即急性和慢性炎性、肝、肾、自身免疫和代谢疾病）和其他恶性疾病患者作为对照队列。对于恶性病变队列，必须进行临床分期（cTNM）或病理分期（pTNM）以及国际抗癌联盟（UICC）分期，并记录转移数量和转移部位。还应记录取样日期、初诊日期、组织学结果、人口数据和吸烟习惯（目前吸烟者、过去吸烟者或从不吸烟者）。

平行测量肌酐与ProGRP，慢性肾脏病流行病学协作组（CKD-EPI）公式被^[33, 34]^用于估算肾小球滤过率（eGFR），以阐明肾功能不全对ProGRP浓度的影响。选择≥0 mL/min/1.73 m^2^（CKD 3期）作为截断值，因为在CKD3期以上患者中ProGRP水平显著升高（补充图S2）。统计分析基于受试者工作特征（ROC）曲线和曲线下面积（AUC）计算，其中CI基于DeLong方法^[35]^。还计算了特异性为95%时的敏感性，以及敏感性为95%时的特异性。

## 4 结果

### 4.1 技术评估

实验室间研究合并样本的CV范围为1.2%-4.9%。约-30 pg/mL和-200 pg/mL合并样本的回收率范围为94%-106%。实验室间的-1, 500 pg/mL样本的回收率变化较大，Bonn中心（104%）和Amsterdam中心（85%）之间的回收率差异大约为20%。

3个欧洲中心人血清池样本和2个对照样本的中间不精密度范围为2.2%（95%CI: 1.8%-2.7%）至6.0%（95%CI: 4.7 % - 8.4 %）CV（补充表S1），与报道的ARCHITECT ProGRP检测（血清和血浆CV为2.2%-5.7%）数据相当^[23]^。批内不精密度范围为1.1%（95%CI: 0.9%-1.4%）至3.0%（95%CI: 2.5%-3.8%）CV。

在来自Amsterdam的9个样本和来自Bonn的10个样本中评价样本稳定性，ProGRP浓度范围为18 pg/mL-4, 259 pg/ mL。使用血浆和血清样本在cobas^®^检测上获得了同样的回收率，当存储在室温下超过4 h（在4 h时的回收率中位数：血浆为98%，SST为102%，RST样本为99%）或在2 ℃-8 ℃下超过48 h（在48 h时的回收率中位数：血浆为97%，SST为101%，RST样本为101%）（图 1）。在-20 ℃下保存12周的血浆和血清样本中也证明了ProGRP良好的稳定性（补充图S3）。相反，使用ARCHITECT检测，血清中ProGRP随时间推移回收率降低（在4 h时的回收率中位数：SST为90%，RST样本为69%），正如之前所报道，血浆和血清样本的结果不相当^[24]^。

**1:**
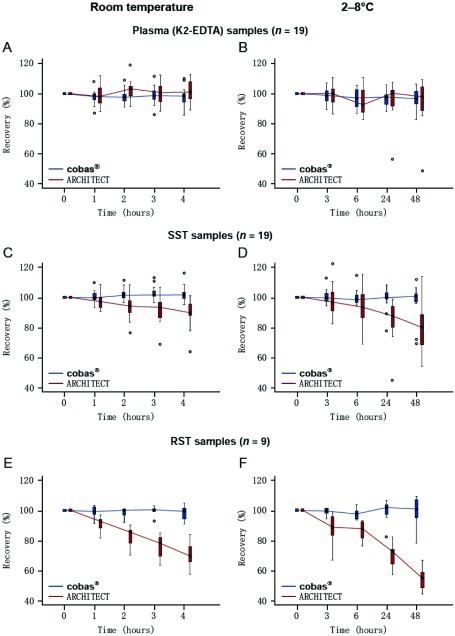
在Amsterdam（*n* =9）和Bonn（*n* =10）获得的患者样本的ProGRP回收率中位率，在室温下孵育超过4 h（A、C、E）或2 ℃-8 ℃下孵育超过48 h（B、D、F），并在cobas^®^或ARCHITECT仪器上测定。 Median ProGRP recovery frompatient samples taken in Amsterdam (*n*=9) and Bonn (*n*=10), incubated at room temperature over 4 hours (A, C, E) or at 2 ℃-8 ℃ over 48 hours (B, D, F) and measured on the cobas^®^ or ARCHITECT instruments.

使用血浆样本的ProGRP浓度高达500 pg/mL，ARCHITECT和cobas^®^检测的相关性如图 2A所示。所有中心合并的斜率和截距分别为1.02（95%CI: 0.96-1.08）和-2.72pg/mL（95%CI: -5.22-0.66），相关系数分别为0.96（补充表S2）。在3 pg/mL-5, 000 pg/mL的整个测定范围内，所有中心报告了类似的发现（数据未显示）。在所有中心，在cobas^®^检测中，ProGRP浓度高达500 pg/mL时，血清和血浆样本间的相关性为：斜率为0.93（95%CI: 0.89-0.98），截距为2.35 pg/mL（95%CI: -0.21-4.60），相关系数为0.97（图 2B）。在Barcelona中心，矩阵比较的斜率显著高于其他中心。其原因未知，但认为与临床无关，并突出了现场检测的变异性。通过使用ProGRP浓度为500 pg/mL的血清样本，可以获得Fujirebio和cobas^®^检测之间相关性的可比结果（图 2c）。在所有中心，斜率为1.33（95%CI: 1.15-1.47），截距为-4.18 pg/ mL（95%CI: -10.50-2.95），相关系数为0.84。

**2:**
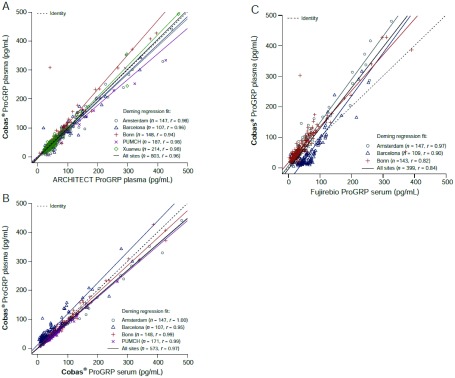
A：方法比较：使用血浆样本的ARCHITECT与cobas^®^比较，直到ProGRP浓度为500 pg/mL；B：矩阵比较：在cobas^®^上的血清与血浆样本比较；C：方法比较：使用血清样本的Fujirebio与cobas^®^比较。 A: Method comparison: ARCHITECT versus cobas^®^ using plasma samples up to a ProGRP concentration of 500 pg/mL; B: Matrix comparison: serum versus plasma samples on cobas^®^; C: Method comparison: Fujirebio versus cobas^®^ using serum samples.

### 4.2 临床评估

来自每个患者队列和每个中心的样本数显示在表 1中。

**1:** 每个中心的每个队列的样本数 Sample numbers for each cohort from each site

队列		Kiel	Amsterdam	Barcelona	Bonn	PUMCH	Xuanwu	所有中心
健康人群	*n*	698	100	100	41	71	75	1, 085
	平均年龄，岁（SD）	49(17)	51(13)	51(7)	38(12)	39(15)	38(12)	48(16)
	男性/女性, *n*	336/362	50/50	33/67	4/37	31/40	24/51	478/607
SCLC	*n*	-	67	68	35	22	15	207
	平均年龄，岁（SD）	-	61(10)	65(12)	62(10)	62(13)	60(8)	62(11)
	男性/女性, *n*	-	38/29	53/15	23/12	17/5	12/3	143/64
	局限期/广泛期*n*	-	38/29	26/42	14/21	9/13	5/10	92/115
NSCLC	*n*	-	397	316	44	42	53	852
	平均年龄，岁（SD）	-	62(12)	67(11)	62(10)	60(10)	65(11)	64(12)
	男性/女性, *n*	-	243/154	229/87	29/15	25/17	28/25	554/298
	腺癌, *n*	-	144	183	15	28	28	398
	鳞状细胞癌, *n*	-	130	91	12	10	20	263
	NSCLCNOS, *n*	-	106	32	16	4	4	162
	大细胞癌, *n*	-	17	10	1	-	1	29
良性肺部疾病	*n*	-	-	28	11	17	41	97
	平均年龄，岁（SD）	-	-	61(13)	61(9)	44(15)	57(14)	56(15)
	男性/女性, *n*	-	-	16/12	3/8	7/10	24/17	50/47
其他良性疾病	*n*	-	-	72	74	16	15	177
	平均年龄，岁（SD）	-	-	47(21)	57(17)	44(18)	62(12)	52(19)
	男性/女性, *n*	-	-	34/38	29/45	9/7	9/6	81/96
其中肾脏疾病	*n*	_	_	27	6	5	1	39
其他恶性疾病	*n*	-	116	100	119	17	15	367
	平均年龄，岁（SD）	-	59(13)	68(13)	63(12)	51(14)	65(11)	63(13)
	男性/女性, *n*	-	58/58	50/50	47/72	12/5	13/2	180/187
其中MCT	*n*	-	11	4	-	-	-	15
其中肺部NET	*n*	-	13	8	-	1	-	22
NOS：未另行规定；MCT：甲状腺髓样癌；NET：神经内分泌肿瘤。 注：本表得到版权所有者©2015 Elsevier B.V.复制许可 Note: Reprinted with permission from the copyright holder ©2015 Elsevier B.V.								

参考范围队列由平均年龄为48岁[标准差（SD）16岁]的1, 085例表观正常人群组成。NSCLC队列包括852例平均年龄64岁（SD 12岁）的患者，SCLC队列由207例平均年龄62岁（SD 11岁）的患者组成。

在参考范围队列中，肝素锂血浆中第95百分位数ProGRP浓度为68 pg/mL（95%CI: 63.7-74.5），EDTA血浆中为60 pg/mL（95%CI: 55.8-65.3），血清样本中为66 pg/mL（95%CI: 62.4-72.6）（图 3）。这些数据与ARCHITECT检测（血清为63 pg/mL；EDTA血浆为65 pg/mL）的参考范围值相当^[36]^，但高于之前报道的Fujirebio检测（血清中43 pg/mL）^[37]^。在欧洲和中国中心之间没有观察到ProGRP浓度中位数的显著差异。

**3:**
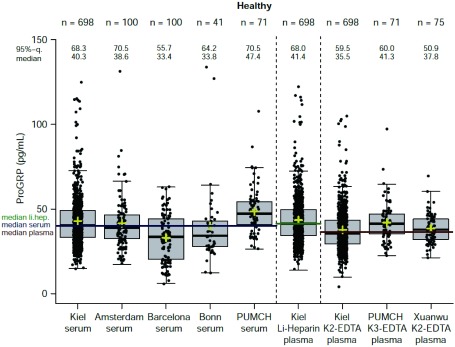
在所有中心健康人群的血清和血浆样本中ProGRP的参考范围分布。黄色十字架代表平均值。 Reference range distribution of ProGRP in serum and plasma samples from healthy individuals for all sites. The yellow crosses represent the mean.

ProGRP在SCLC和NSCLC之间表现出良好的鉴别诊断能力，在NSCLC队列中AUC为0.90（95%CI: 0.87-0.93），敏感性为78.3%，特异性为95%。ProGRP的分布如图 4所示。虽然SCLC队列中的ProGRP浓度在中国中心中趋于更高（数据未显示），但是欧洲和中国中心的NSCLC和SCLC队列中没有观察到临床相关的中心效应。未注意到年龄、性别或吸烟习惯对ProGRP浓度的临床相关影响（数据未显示）。基于NSCLC队列的95%特异性，将SCLC（*n*=207）与NSCLC（*n*=852）区分的临床鉴别诊断截断值计算为84 pg/mL（95%CI: 76.9-98.8）。

**4:**
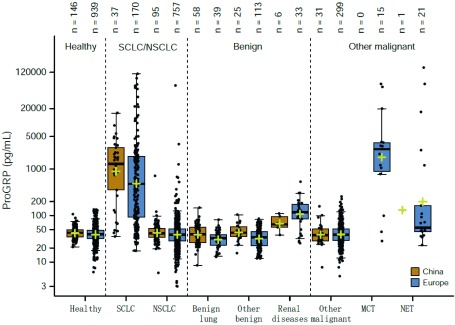
中国和欧洲中心不同临床队列的ProGRP浓度。黄色十字架代表平均值。 ProGRP concentrations in different clinical cohorts in China and Europe. The yellow crosses represent the mean.

ProGRP值在局限期与广泛期SCLC患者的样本中具有差异（中位数分别为351 pg/mL和757 pg/mL；*P*=0.002, 3）（补充图S4）。在NSCLC队列中95%的固定特异性下，鉴别局限期SCLC与NSCLC的敏感性为71.7%（AUC为85.6；95%CI: 80.3-90.9），鉴别广泛期SCLC的敏感性为83.5%（AUC为93.0；95%CI: 89.8-96.3）。在SCLC患者中，虽然Ⅰ期和Ⅱ期SCLC患者的样本量较少（数据未显示），但UICC疾病分期与ProGRP水平之间的相关性显著。在所有中心，ProGRP在除外肾脏疾病的良性疾变患者（中位数为38 pg/mL），以及除外肾脏疾病，肺MCT或NET的其他恶性肿瘤患者（中位数为40 pg/mL）中浓度较低。与3个欧洲中心[AUC 0.89（95%CI: 0.85-0.92）；NSCLC队列中特异性为95%时敏感性为76.9%）]相比，在两个中国中心[AUC 0.94（95%CI: 0.89-0.99）；特异性为95%时敏感性为83.8%]中，ProGRP显示出更高的SCLC和NSCLC鉴别诊断能力，虽然不具有统计学意义，可能是由于中国队列的样本量较少（图 5）。

**5:**
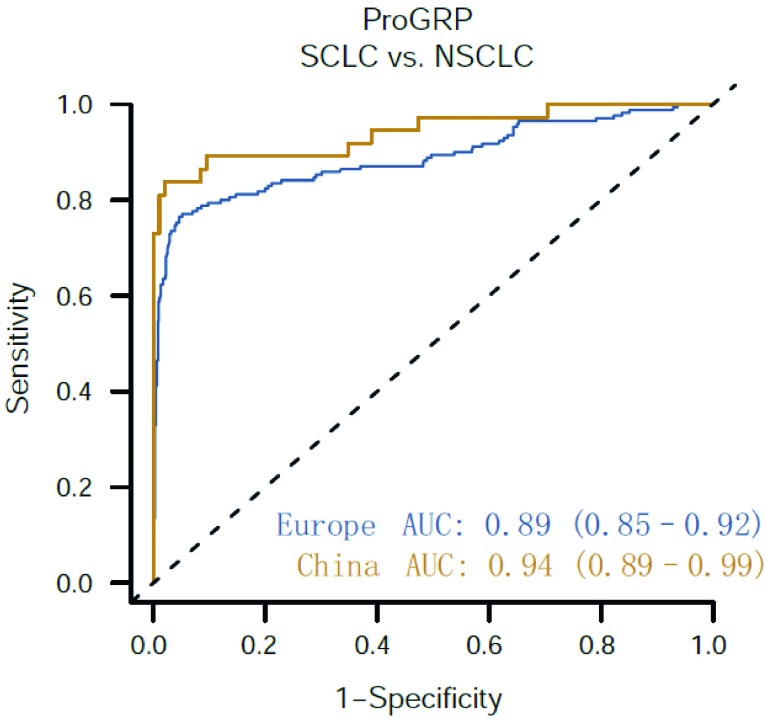
欧洲和中国中心ProGRP（eGFR≥30mL/min/1.73 m^2^）用于SCLC与NSCLC相比的ROC曲线。欧洲SCLC（*n*=170），NSCLC（*n*=757）；中国SCLC（*n*=37），NSCLC（*n*=95）。 ROC curves of ProGRP (eGFR≥30 mL/min/1.73 m^2^) for SCLC versus NSCLC for European and Chinese sites. Europe SCLC (*n*=170), NSCLC (*n*=757); China SCLC (*n*=37), NSCLC (*n*=95).

## 5 讨论

在多个欧洲和中国中心进行的Elecsys^®^ ProGRP检测的多中心评估显示出良好的不精密度、稳定性和特异性。中间不精密度值范围为CV：2.2%-6.0%，与ARCHITECT ProGRP检测报道的数据相当^[23]^。批内不精密度范围为CV：1.1%-3.0%。值得注意的是，Elecsys^®^ ProGRP检测在EDTA血浆和血清样本中等同进行，在一定范围的储存条件下回收率保持不变。存储样本并不总是被认为与新鲜样本一样可靠，但我们的数据表明ProGRP在存储和新鲜组织中均保持稳定。相比之下，血清中ProGRP的回收率随ARCHITECT检测时间下降，正如之前所报道的，在血浆和血清样本中获得不同的结果^[24]^。

研究报告的ARCHITECT检测分析的血清样本相关的稳定性问题，研究认为部分归因于血清样本中的凝血酶，因此血浆被鉴定为首选源材料^[24]^。相对于ARCHITECT检测，Elecsys^®^ ProGRP检测在血清中具有较好的稳定性，最有可能与ProGRP肽上的抗体结合位点有关。Elecsys^®^ ProGRP检测中的两个单克隆抗体与耐内切蛋白酶酶切的表位结合^[16, 17, 26]^，而ARCHITECT检测中的两个单克隆捕获抗体直接结合凝血酶酶切位点（补充图S1）。

在ProGRP浓度高达500 pg/mL（斜率1.02，截距-2.72 pg/mL）所有中心中，并在整个测定范围内，ARCHITECT和cobas^®^检测在血浆中均显示出良好的相关性。同时证实在cobas^®^检测中，ProGRP浓度高达500 pg/mL（斜率为0.93，截距为2.35 pg/mL）时，并在整个测定范围内，血清和血浆之间表现出良好的相关性。在所有中心，ProGRP浓度高达500 pg/mL（斜率为1.33，截距为-4.18 pg/mL）的血清样本中，Fujirebio和cobas^®^检测之间也存在良好的相关性。值得注意的是，Bonn和Barcelona实验室的样本没有均匀地涵盖测量范围，该范围内大多数样本的ProGRP浓度均低于500 pg/mL。ARCHITECT和cobas^®^检测在整个测定范围方面的差异小于ARCHITECT和Fujirebio ProGRP检测（斜率为0.93，Passing-Bablok回归；相关系数为0.99）之间相关性的差异^[36]^。

在Elecsys^®^ ProGRP检测中，所测定的表观正常人群的ProGRP参考范围与ARCHITECT检测的参考范围的结果相当^[36]^。来自Barcelona和Bonn的样本中的ProGRP浓度比其他中心的略低。

ProGRP在血清中显示出明确的SCLC和NSCLC鉴别诊断能力，在NSCLC队列中，特异性为95%时，敏感性为78%，如先前所述ARCHITECT ProGRP检测的结果^[16, 17, 21, 36]^。在NSCLC和SCLC队列中，各中心间无临床相关差异。此外，未见年龄、性别或吸烟习惯的临床相关影响。本研究为专门针对中国患者的ProGRP检测的首次评价，并且证明了不同种族之间的结果的重复性。

尽管早期SCLC患者的数量很少，如先前研究所报道的，与局限期SCLC患者相比，广泛期SCLC患者的ProGRP浓度较高^[25, 38]^。此外，研究还发现肿瘤大小（根据UICC分期）与ProGRP浓度之间存在相关性。正如研究所预期，NSCLC、良性肺病、其他良性疾病或其他恶性肿瘤（不包括肾脏疾病、肺的MCT和NET）患者的ProGRP浓度较低。因为仅发现来自肺或原发灶未知的NET患者的ProGRP水平升高^[22]^，ProGRP可能是定位原发部位未知NET的有效诊断工具。在所有欧洲和中国中心，eGFR≥30 mL/min/1.73 m^2^（CKD 3期）患者血清中，ProGRP对于SCLC和NSCLC均表现出良好的鉴别诊断能力，但在eGFR < 30 mL/min/1.73 m^2^患者中应注意解读ProGRP结果。

许多研究中已经确立了ProGRP作为生物标志物鉴别SCLC与其他类型肺癌的临床应用^[12, 16, 20, 21, 39-42]^。在对11项临床试验中纳入的5146例患者进行的荟萃分析中，ProGRP在SCLC诊断中的敏感性和特异性分别为0.716（95%CI: 0.688-0.743）和0.921（95%CI: 0.909-0.932）^[40]^。Elecsys^®^ ProGRP检测的临床表现与这些结果完全一致。该Elecsys^®^ProGRP检测多中心评估的数据表明，ProGRP是SCLC的特异性肿瘤标志物，可用于肺癌的鉴别诊断。Elecsys^®^ProGRP检测在血清中的稳定性增加，在临床实验室常规使用中具有明显的优势，因为血清是肿瘤标志物检测的首选样本。目前正在进行一项对ProGRP在治疗监测中的应用研究，以及在相同采集样本中测定和分析其他肿瘤标志物（CEA、NSE、CYFRA 21-1）的研究。

本文的补充数据可以在网上找到（http://dx.doi.org/10.1016/j.cca.2014.09.015.）
